# Optimization of Contrast Injection Protocols for Time‐Resolved MRA Technique in Dogs: A Comparative Study of Vascular Signal Characteristics and Artifacts

**DOI:** 10.1111/vru.70107

**Published:** 2026-01-13

**Authors:** Sunghwa Hong, Eunji Kim, Junghee Yoon, Jihye Choi

**Affiliations:** ^1^ Department of Veterinary Medical Imaging College of Veterinary Medicine and Research Institute for Veterinary Science Seoul National University Seoul South Korea; ^2^ NEL Animal Medical Center Anyang‐si Gyeonggi‐do South Korea

**Keywords:** artifact, canine, computed tomography angiography (CTA), dog, magnetic resonance imaging (MRI)

## Abstract

This study aimed to optimize magnetic resonance angiography (MRA) protocols for time‐resolved MRA imaging in dogs by using different injection rates and contrast volumes. In this experimental and prospective study, four protocols combining two flow rates (0.2 and 2.0 mL/s) and two contrast volumes (0.2 and 0.4 mL/kg, equivalent to 0.1 and 0.2 mmol/kg gadolinium) were applied in five healthy beagle dogs. Quantitative measurements, including maximum signal intensity, peak enhancement time, diagnostic window, and signal homogeneity, were obtained for the common carotid artery and external jugular vein. Qualitative assessment included arterial visibility persistence, wall margin clarity, and artifact evaluation. Statistical comparisons were performed using the Friedman and Wilcoxon signed‐rank tests, and effect size analysis was used to further interpret nonsignificant trends. The low‐flow–high‐volume protocol (0.2 mL/s, 0.4 mL/kg) yielded the longest diagnostic window and superior vessel visibility with minimal venous contamination, while maintaining comparable maximum signal intensity to higher flow rate protocols. Artifact‐related issues such as ringing and intravoxel dephasing were least observed in the low‐flow–high‐volume protocol. Although high‐flow–high‐volume protocol showed improved signal homogeneity, it was associated with greater artifact susceptibility. Interobserver agreement ranged from fair to substantial (*κ* = 0.457–0.681), with greater variability in artifact‐related scores. These findings suggest that a slower injection rate with higher contrast volume, as seen in the low‐flow–high‐volume protocol, provides the best balance of image quality and diagnostic performance, supporting its recommendation as a preferred protocol for small‐animal time‐resolved MRA.

**Abbreviations**
ARRIVE: Animal Research: Reporting of In Vivo Experiments
CCA: common carotid artery
CE‐MRA: contrast‐enhanced magnetic resonance angiography
CTA: computed tomography angiography
CV: coefficient of variation
dwMRA: diagnostic imaging window for magnetic resonance angiography
EJV: external jugular vein
FOV: field of view
HR: heart rate
IQR: interquartile range
MAP: mean arterial pressure
maxSNR: maximum signal‐to‐noise ratio
MIP: maximum intensity projection
MRA: magnetic resonance angiography
MRI: magnetic resonance imaging
PACS: picture archiving and communication system
ROI: region of interest
SD: standard deviation
SNR: signal‐to‐noise ratio
SPSS: Statistical Package for the Social Sciences
TRICKS: time‐resolved imaging of contrast kinetics

## 1 Introduction

With advances in medical imaging technology, contrast agents have been continuously developed to improve image quality, and diverse imaging techniques utilizing these agents have evolved in parallel. A central principle of contrast‐enhanced imaging is the comparison between pre‐ and post‐contrast acquisitions, and the methodology of contrast administration—particularly the route, timing, and rate of injection—has become a key determinant of image quality and diagnostic performance. These considerations are especially critical in vascular imaging, where precise timing of contrast passage is essential for clear arterial visualization.

In veterinary medicine, vascular imaging is primarily performed using computed tomography angiography (CTA) and magnetic resonance angiography (MRA) [1]. Contrast‐enhanced MRA (CE‐MRA) offers improvements over non‐contrast techniques, which are limited by complex tissue contrast, artifact susceptibility, and prolonged scan times [2]. Nevertheless, CE‐MRA is constrained by delayed acquisition relative to the advancing contrast bolus, often leading to venous contamination and suboptimal arterial visualization. Efforts to mitigate this with rapid injections may, in turn, cause abrupt intravascular signal fluctuations and *k*‐space artifacts, thereby compromising overall image quality [3].

Time‐resolved MRA was developed to address the limitations of conventional CE‐MRA by acquiring rapid sequential images during contrast transit through the vasculature. This technique is accomplished by more frequently sampling the central region of *k*‐space—where contrast information is most prominent—more frequently than the periphery, while sharing peripheral *k*‐space data between adjacent frames via a view‐sharing algorithm. This asymmetric *k*‐space acquisition, often combined with elliptical centric ordering and parallel imaging methods, allows for sub‐second temporal resolution without compromising spatial resolution. This technique improves temporal resolution, reduces the risk of mistimed acquisitions, and allows reliable differentiation of arterial and venous phases. Nevertheless, compared with conventional CE‐MRA, time‐resolved MRA generally has lower spatial resolution and provides less detail regarding the anatomical relationships between vessels and adjacent structures. These trade‐offs highlight the importance of evidence‐based optimization of contrast injection protocols, which remain largely undefined in dogs.

Although injection parameters, such as rates and volumes, have been extensively studied in computed tomography (CT), comparable data for MRA in canine patients are limited [4, 5, 6]. In human studies, the effects of injection rate and contrast dose on vascular imaging quality have been extensively investigated [7, 8]. Rapid contrast injections can achieve sharp arterial enhancement but may produce abrupt intravascular signal variability and blooming artifacts [9]. In contrast, slow injections may yield inadequate arterial enhancement and poor signal‐to‐noise ratio (SNR) [10]. Similarly, excessive contrast doses can result in *T*2* effects, signal saturation, and venous contamination, whereas insufficient doses cause weak vessel conspicuity and diminished diagnostic confidence [7, 11]. Because of the substantial differences between humans and dogs—including wide variation in body size, smaller body mass, and physiological differences in heart rate (HR), cardiac output, and circulation time—contrasting administration protocols established in human medicine may not be directly applicable to veterinary patients. Therefore, individualized optimization of both injection rate and dose is critical for obtaining high‐quality, artifact‐free MRA images in veterinary patients.

In the optimization of time‐resolved MRA, minimizing artifacts is crucial, particularly with respect to ringing artifacts known as Maki artifacts and pseudostenosis. Ringing artifacts appear as a double contour of the vessel wall with a central zone of low signal intensity. They occur when the central portion of *k*‐space, which contains most of the contrast and signal intensity information, is acquired too early—before the contrast agent has adequately reached the vessel [12, 13]. Because the peripheral *k*‐space encodes fine structural detail, this temporal mismatch can reduce vascular enhancement and distort vessel wall delineation. Such *k*‐space sampling limitations, together with other technical and anatomical factors, may also lead to pseudostenosis. CE‐MRA is known to overestimate vascular stenosis due to limited spatial resolution, suboptimal bolus timing, and phase‐related signal loss such as intravoxel dephasing [14]. These factors are particularly relevant in time‐resolved MRA, which employs view sharing and temporal undersampling to enhance temporal resolution.

This study was designed to optimize time‐resolved MRA protocols in dogs, with a particular focus on the intracranial and cervical vasculature. Because the intracranial arteries and veins of dogs are small in caliber and provide insufficient signal for accurate measurement, the common carotid artery (CCA) and external jugular vein (EJV) were selected as target vessels due to their relatively large diameter, strong and consistent signal, and close hemodynamic relationship with intracranial circulation. These vessels are directly connected to clinically relevant vascular territories and therefore provide meaningful information in conditions such as ischemic and hemorrhagic cerebrovascular disease, congenital vascular malformations, and head and neck tumors with intra‐ and peritumoral neovascularization. Using this model, multiple injection protocols with varying rates and contrast volumes were applied to normal dogs, analyzing their effects on arterial enhancement, SNR, and overall image quality. The ultimate objective was to determine the optimal combination of injection rate and contrast dose that would maximize image quality while minimizing artifacts.

This study hypothesized that in canine time‐resolved MRA, (1) the injection rate significantly influences arterial enhancement and SNR, (2) the injection volume affects both vascular conspicuity and venous contamination, and (3) an optimized combination of rate and volume provides superior arterial–venous separation and minimizes common CE‐MRA artifacts, thereby maximizing overall diagnostic image quality.

## 2 Materials and Methods

This study was approved by the Institutional Animal Care and Use Committee of Seoul National University, and the animals were cared for in accordance with the Guidelines for Animal Experiments of Seoul National University (SNU‐241211‐1). Furthermore, this study adhered to the Animal Research: Reporting of In Vivo Experiments (ARRIVE) guidelines to ensure ethical and transparent reporting of animal experiments in line with international standards.

### 2.1 Animals

This study was designed as an experimental and prospective investigation, involving five purpose‐bred adult Beagle dogs. The sample size was limited to five animals for two main reasons: first, to assess intra‐individual variability under identical experimental conditions; and second, due to the inherent challenges of magnetic resonance imaging (MRI) examinations, which require prolonged anesthesia, high costs, considerable time, and substantial personnel involvement. The cohort consisted of three males and two females, with a mean age of 4 years (range: 3–5 years) and an average body weight of 13.8 kg (range: 11–15 kg). All dogs were deemed clinically healthy on the basis of comprehensive physical examinations, blood pressure and HR assessments, complete blood counts, serum biochemistry profiles, urinalysis, thoracic and abdominal radiography, abdominal ultrasonography, and echocardiography. None of the animals had a history or clinical signs of neurological disorders.

### 2.2 Anesthesia

After an 8‐h fast, a 24‐gauge catheter was inserted into the cephalic vein, and anesthesia was induced via intravenous injection of 0.1 mg/kg medetomidine (Domitor) and 2.0 mg/kg alfaxalone (Alfaxan, Jurox Pty Ltd., Rutherford, NSW, Australia) in each dog. Then, anesthesia was maintained with sevoflurane (Sevofran, Hana Pharm, Korea) and oxygen (1–1.2 L/min) via an endotracheal tube. The dogs were maintained under positive pressure ventilation using 100% oxygen with a peak inspired pressure of 10–12 mmHg at a respiratory rate of 10–28 breaths/min, tidal volume of 15 mL/kg, and end‐tidal CO_2_ concentration of 35–45 mmHg. During the anesthesia, HR, end‐tidal carbon dioxide, body temperature, and noninvasive blood pressure were monitored. HR and blood pressure were monitored approximately every 5 min. After completing the imaging procedures, anesthesia was discontinued, and the dogs were monitored closely during recovery. The endotracheal tube was removed once the palpebral reflex, gag reflex, and the ability to lift their head had returned. The dogs were discharged to the facility once they were fully alert and able to stand unassisted. During the recovery period, HR, respiratory rate, and body temperature were continuously monitored to ensure stability. Anesthesia was performed a total of four times per dog in five dogs over a period of approximately 3 weeks. All potential adverse effects associated with anesthesia were carefully assessed.

### 2.3 MRA Protocol

Underwent general anesthesia, the dog was placed in the sternal recumbency position with the head first in the scanner. All MRI scans were performed using a 1.5‐T magnet (SIGNA Creator; GE Healthcare, Milwaukee, WI, USA) with an 8‐channel flex coil. Three orthogonal plane images of 15–16 cm of field of view (FOV) were obtained with *T*2‐weighted MRI. Then, time‐resolved imaging of contrast kinetics (TRICKS) sequence was performed using dorsal plane.

The TRICKS sequences were performed with the following parameters: repetition time of 5.3 ms, echo time of 2 ms, and a flip angle of 50°. The FOV was set to 280 × 280 cm^2^, with the phase FOV adjusted to 60% of the frequency FOV. The matrix size was 384 × 256, and the voxel size was 0.7 × 1.1 × 2 mm^3^. The bandwidth was set to 62.5 MHz, with a slice thickness of 2 mm and the number of excitations set to 1. Imaging was performed in the dorsal plane, ranging dorsally from the transverse sinus of the intracranial venous system to ventrally the EJV. A total of 42–44 planned slices were acquired per subject. The mask was acquired over approximately 18–20 s prior to the temporal post‐contrast acquisition. Following contrast administration, a minimal scan delay (about 1 s) was applied, after which a total of 48 phases were obtained. The scan was initiated concurrently with the contrast injection. Each phase had a scan time between 4 and 5 s. The frequency‐encoding direction was dorsal/ventral. A paramagnetic gadolinium‐based contrast agent (Dotarem, Guerbet, France) was administered via a cephalic vein catheter using an automatic dual power injector (OptiStar Elite, Mallinckrodt, Missouri, USA) with a Y‐shaped extension directly connected to the intravenous catheter, without any additional extension line.

In each dog, four different MRA protocols were applied using combinations of two flow rates (0.2 and 2.0 mL/s) and two contrast agent volumes (0.2 mL/kg [0.1 mmol/kg] and 0.4 mL/kg [0.2 mmol/kg]): low flow–low volume (0.2 mL/s, 0.2 mL/kg), low flow–high volume (0.2 mL/s, 0.4 mL/kg), high flow–low volume (2 mL/s, 0.2 mL/kg), and high flow–high volume (2 mL/s, 0.4 mL/kg). The saline volume (10 mL) and saline flow rate (2 mL/s) were kept constant across all protocols. The order of the four injection protocols was determined using a random number generator (Microsoft Excel), with each protocol separated by at least a 48‐h interval to ensure complete clearance of the contrast agent in each dog. The acquisition time for TRICKS was recorded from the start of post‐contrast imaging to the end of the sequence, excluding the mask acquisition time. After subtraction processing using baseline images, maximum intensity projection (MIP) was generated. The image for each time phase was combined into a single series.

### 2.4 MRA Image Analysis

All MRA images were sent to a picture archiving and communication system (Infinitt PACS; Infinitt Healthcare, Seoul, South Korea) for analysis. The vascular analysis was performed on the CCA, the largest artery in diameter among the major arteries leading to the head, and the EJV, the main channel for the return of venous blood from the head.

### 2.5 Quantitative Assessment of MRA

**FIGURE 1:**
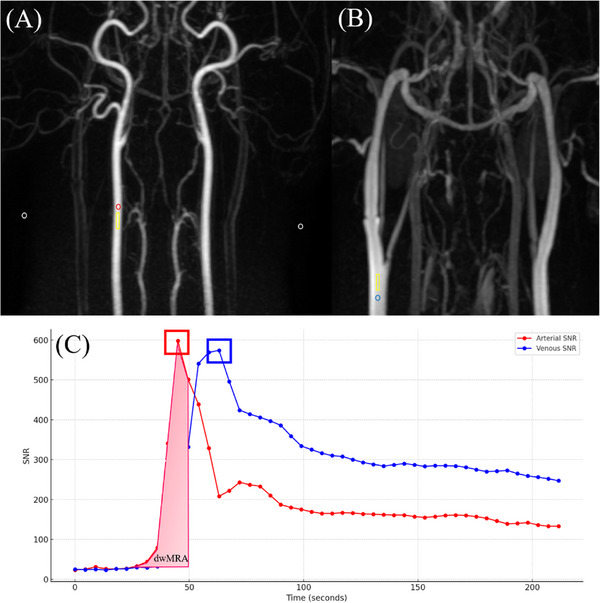
Dorsal images from low‐flow–high‐volume protocol using time‐resolved imaging of contrast kinetics with maximum intensity projection reconstruction. (A) Phase 11 and (B) Phase 15. A red circular region of interest (ROI) for signal intensity was placed at the center of the right common carotid artery in (A), and a blue ROI of identical size was placed at the center of the right external jugular vein in (B). To measure the standard deviation of background noise, identical circular ROIs (white circles) were placed in the air regions on both the left and right sides in (A). A rectangular ROI (yellow rectangle) was also placed at the same level to measure the coefficient of variation. (C) Time–intensity curves generated from circular ROIs placed within the artery and vein. The red and blue lines represent the arterial and venous signal‐to‐noise ratio (SNR) values, respectively. Colored squares indicate the peak SNR values for each vessel. The diagnostic imaging window for MRA (dwMRA), illustrated as a shaded light red area, corresponds to the area under the arterial SNR area under curve up to the point just before the venous SNR surpasses the arterial SNR.

The quantitative assessment was performed for vascular enhancement, diagnostic imaging window, and vascular signal homogeneity across different MRA protocols in TRICKS sequences. For analysis, region of interest (ROI)‐based measurements were performed using the time–intensity curve (TIC) function available in the PACS software, which displays sequential intensity values over time in a graphical format (Figure 1). For arterial evaluation, a circular ROI (diameter, 1–2 mm) was drawn as centrally as possible within the lumen of the CCA, prior to its bifurcation into the internal and external carotid arteries, based on the MIP images. For venous evaluation, a similarly sized circular ROI was placed within the EJV at the level before its bifurcation into the maxillary and linguofacial veins. To assess background noise, two separate ROIs of the same size were placed in air regions adjacent to the skin, as background noise is not uniform in parallel imaging.

For evaluating vascular enhancement, SNR was calculated by dividing the measured signal intensity by the average of the standard deviations (SDs) obtained from the two background noise ROIs. Among the obtained values, the highest SNR within each dataset was defined as the maximum signal‐to‐noise ratio (maxSNR), representing the peak signal intensity to background variation during contrast enhancement. The number of phases required to reach the maxSNR was multiplied by the scan time per phase to calculate the peak enhancement time.

In this study, the diagnostic imaging window for MRA (dwMRA) was defined in a simplified manner as the arterial area under the curve (AUC) measured until the venous SNR exceeded the arterial SNR. Unlike the definition proposed by Du et al., we did not subtract a venous AUC component, and thus, our dwMRA represents only the usable arterial signal prior to venous overlap [7, 15].

Vascular signal homogeneity was assessed by measuring the coefficient of variation (CV) of intravascular signal intensities [16]. Rectangular ROIs, approximately 1–2 mm (short axis) × 4–6 mm (long axis), were manually drawn parallel to the vessel axis and as centrally as possible within the vascular lumen. The ROIs were placed at the peak enhancement phase of both arterial and venous structures, as determined from the TIC, and with appropriate adjustment of window levels to identify the areas of maximum signal intensity. The CV, defined as the ratio of the SD to the mean signal intensity within each ROI, was used to represent the relative distribution and variability of signal intensities.

All evaluations were performed by one investigator (H.S.H.), a PhD candidate in veterinary medical imaging with extensive training and 10 years of experience in advanced imaging techniques. The evaluation parameters, including the selection of ROIs and measurement methods, were discussed and finalized under the supervision of a diplomate of the Korean College of Veterinary Medical Imaging (J.H.C). to ensure consistency and accuracy.

### 2.6 Qualitative Assessment of MRA

Qualitative evaluation of MRA image quality was independently and blindly performed by two reviewers: one (H.S.H), a PhD candidate in veterinary medical imaging and the other (K.E.J.), a third‐year doctoral student in the same field. The image sets were presented to each reviewer in random order, blinded to MRA protocols and to the evaluations of the other reviewer. The assessments were conducted using dorsal MIP reformatted images, with scrolling through the entire dataset to comprehensively examine the temporal and spatial characteristics of the vessels.

For the qualitative assessment, total six evaluation factors related with the arterial clarity, vessel visibility, and image quality were assessed and subjectively scored using a 3‐point scale: 1 indicated poor, 2 indicated intermediate, and 3 indicated optimal image quality (Table 1). Arterial clarity was assessed by evaluating venous contamination, defined as the degree to which the CCA could be distinguished from the adjacent EJV during the arterial phase. The vessel visibility evaluation included vessel delineation and smoothness of vessel margins. The temporal vessel delineation was assessed by determining whether the vascular structures remained distinguishable from surrounding tissues from the peak enhancement phase through to the final acquired phase. The smoothness of vessel margins was evaluated on the basis of the uniformity and clarity of the vessel wall margins at peak contrast enhancement, assessed at the level of the CCA. For image quality, three artifacts were assessed. Ringing artifacts in the CCA were assessed by evaluating the presence of repetitive signal oscillations along the vessel wall during the arterial phase, commonly resulting from contrast bolus misregistration or susceptibility differences. Ringing artifacts in the EJV were assessed by evaluating the presence of repetitive signal oscillations along the vessel wall during the venous phase. Ringing artifacts were defined as those appearing as a double contour of the vessel wall with a central zone of low signal intensity within the vessel. Pseudostenosis or asymmetrical vascular signal loss caused by intravoxel dephasing artifacts was assessed with attention to regions mimicking true stenosis due to magnetic susceptibility effects or flow‐related dephasing.

**TABLE 1:** Evaluation criteria of the qualitative assessment factors in time‐resolved imaging of contrast kinetics.

Evaluation factors	Parameters	Criteria	Score
Arterial clarity	Venous contamination	The number of temporal phases that clearly demonstrated the CCA with defined contours and higher signal intensity than the EJV	Less than one phase Two phases More than three phases
Vascular visibility	Temporal vessel delineation	Distinguishability of vascular structures from surrounding tissues throughout the contrast‐enhanced phases, from the contrast peak phase to the final acquired phase	Indistinct vessels in most time points Generally good, but indistinguishable vessels in some frames Clearly delineated vessels throughout all time frames
	Smoothness of vessel margins	Appearance of vessel wall margins at the time of peak enhancement	Highly irregular margins Mild irregularities or minor blurring Smooth and sharply defined margins
Image quality	Ringing artifacts in the CCA	Presence of repetitive signal oscillations along the CCA wall during the arterial phase	Artifacts present in most phases Artifacts present in a few phases Minimal or no artifacts
	Ringing artifacts in the EJV	Presence of repetitive signal oscillations along the EJV wall during the venous phase	Artifacts present across all venous phases Artifacts present in a few phases Minimal or no artifacts
	Pseudostenosis or asymmetric vascular signal loss	Presence of pseudostenotic segments caused by intravoxel dephasing artifacts	Two or more pseudostenotic segments One pseudostenotic segment No pseudostenotic segment

Abbreviations: CCA, common carotid artery; EJV, external jugular vein.

### 2.7 Statistical Analyses

Statistical analysis was performed using Statistical Package for the Social Sciences (SPSS) for Windows (version 29.0; SPSS Inc., Chicago, IL, USA) with guidance from a research professor (E.Y.K.) in medical statistics at a human university hospital.

For comparisons among the four MRA protocols, monitoring data of pre‐anesthesia and intra‐anesthesia, the maxSNR of the CCA and EJV, peak enhancement time, dwMRA, CV values, and qualitative assessment scores were analyzed using the Friedman test with post hoc Wilcoxon signed‐rank tests. For parameters in the quantitative evaluation that showed statistically significant differences in the Friedman test, additional between‐group comparisons were performed using the Mann–Whitney *U* test to assess inter‐protocol variability while accounting for inter‐individual effects. The effect size was calculated using the rank‐biserial correlation (*r*) for post hoc Wilcoxon signed‐rank tests. The magnitude of effect size was interpreted on the basis of common conventions: small (*r* ≥ 0.1), medium (*r* ≥ 0.3), and large (*r* ≥ 0.5) [17]. Interobserver agreement for the qualitative assessments was evaluated using the kappa correlation test. A *p* value of <0.05 was considered statistically significant.

### 2.8 Use of Artificial Intelligence Tools

Portions of the manuscript were edited for language clarity using ChatGPT (OpenAI, San Francisco, CA, USA). All AI‐assisted text was reviewed and verified by the authors to ensure accuracy. No AI tools were used for data analysis, interpretation, or generation of scientific content.

## 3 Results

All dogs completed the time‐resolved MRA sequence without adverse effects related to anesthesia or contrast administration. Mean, SD, and median values for intra‐anesthetic HR and mean arterial pressure (MAP) are summarized in Table 2.

**TABLE 2:** Heart rate and mean arterial pressure measured during anesthesia in five beagle dogs undergoing four magnetic resonance angiography protocols combining two flow rates (0.2 and 2.0 mL/s) and two contrast agent volumes (0.2 and 0.4 mL/kg).

Monitoring data	Low flow–low volume (0.2 mL/s, 0.2 mL/kg)	Low flow–high volume (0.2 mL/s, 0.4 mL/kg)	High flow–low volume (2.0 mL/s, 0.2 mL/kg)	High flow–high volume (2.0 mL/s, 0.4 mL/kg)
**HR during anesthesia**				
Mean ± SD	86.3 ± 21.19	70.6 ± 17.4	83.7 ± 23.4	75.9 ± 26.0
Median (IQR)	88 (66.25–96.5)	70.5 (64.15–74.25)	78.5 (72.34–88)	78 (59–91.5)
**MAP during anesthesia**				
Mean ± SD	98.8 ± 14.4	103.3 ± 20.2	108.2 ± 11.9	109.8 ± 15.8
Median (IQR)	94.5 (85–106)	101 (90–118.5)	109.5 (97–114.75)	110.5 (96–116.75)

*Note*: All data are presented as mean ± standard deviation (SD) and median (IQR). Pairwise statistical comparisons were performed using the Wilcoxon signed‐rank test.

Abbreviations: HR, heart rate; IQR, interquartile range; MAP, mean arterial pressure; SD, standard deviation.

### 3.1 Quantitative Assessment of Image Quality

Considering the small sample size and variability in data distribution, nonparametric statistical methods were used for all analyses. The quantitative assessment of image quality is presented in Table 3. Quantitative analysis demonstrated that injection rate had a significant effect on arterial peak enhancement time, whereas contrast volume affected vascular delineation and CV. In contrast, neither parameter exerted a significant influence on maxSNR.

**TABLE 3:** Quantitative assessment of arterial and venous imaging parameters across four magnetic resonance angiography protocols using the time‐resolved imaging of contrast kinetics.

Parameters	Low flow–low volume (0.2 mL/s, 0.2 mL/kg)	Low flow–high volume (0.2 mL/s, 0.4 mL/kg)	High flow–low volume (2.0 mL/s, 0.2 mL/kg)	High flow–high volume (2.0 mL/s, 0.4 mL/kg)
MaxSNR of CCA (IQR)	420 (321–435)^a^	598 (408–661.5)^a^	313 (283–460)^a^	570 (409–901)^b^
MaxSNR of EJV (IQR)	404 (335–575)^a^	404 (335–575)^a^	309 (222.5–343.5)^a^	418 (334.5–617)^b^
Peak enhancement time of CCA (s)^*^	36 (34.3–38.05)^a^	49.5 (46.8–55.1)^b^	24 (22.5–28)^c^	23.5 (23.5–28.45)^c^
Peak enhancement time of EJV (s)^*^	52 (44.1–53.6)^a^	67.5 (61.1–69)^b^	33.6 (31.5–44.55)^c^	32.9 (32.9–42.75)^c^
dwMRA^*^	4882.85 (4092.60–5802.93)^a^	8098.1 (6406.63–11,120.93)^b^	3314.25 (2451.08–6045.50)^a^	6474.25 (5479.03–7546.95)^b^
CV of CCA	3.35 (2.33–6.27)^a^	2.36 (1.87–3.39)^a^	3.02 (2.07–6.19)^a^	1.13 (0.50–3.62)^b^
CV of EJV	3.55 (2.52–9.23)	2.26 (2.07–3.56)	3.76 (2.69–4.96)	2.19 (1.39–2.71)

*Note*: All data are presented as medians with interquartile ranges. Pairwise statistical comparisons were performed using the Wilcoxon signed‐rank test. Within each row, values with different superscript letters (a–c) indicate statistically significant differences between protocols (*p* < 0.05), as determined by the Wilcoxon signed‐rank test.

In some rows, superscript letters are provided despite a non‐significant Friedman test result. These are based on post hoc Wilcoxon signed‐rank tests and should be interpreted with caution.

Abbreviations: CCA, common carotid artery; CV, coefficient of variation; dwMRA, diagnostic imaging window for magnetic resonance angiography; EJV, external jugular vein; IQR, interquartile range; maxSNR, maximum SNR; SNR, signal‐to‐noise ratio.

* The Friedman test showed a significant difference among protocols (*p* < 0.05).

**FIGURE 2:**
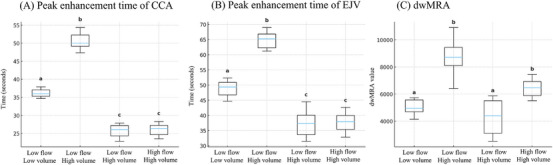
Box plots of parameters showing statistically significant inter‐protocol differences in time‐resolved magnetic resonance angiography (MRA). Peak enhancement times of both the common carotid artery (CCA) (A) and external jugular vein (EJV) (B) were significantly shorter in high‐flow protocols compared to low‐flow protocols, regardless of contrast volume. Within the low‐flow protocols, the low‐volume protocol showed a shorter peak enhancement time than the high‐volume protocol. The diagnostic imaging window for MRA (dwMRA) (C) was significantly larger in the low‐flow–high‐volume and high‐flow–high‐volume protocols compared to the low‐flow–low‐volume and high‐flow–low‐volume protocols. Protocol definitions: low flow–low volume (0.2 mL/s, 0.2 mL/kg), low flow–high volume (0.2 mL/s, 0.4 mL/kg), high flow–low volume (2.0 mL/s, 0.2 mL/kg), and high flow–high volume (2.0 mL/s, 0.4 mL/kg). Boxes represent the interquartile range, whiskers the minimum and maximum values, and the horizontal line within each box the median. Superscript letters denote groups that differ significantly (Friedman test with post‐hoc Wilcoxon signed‐rank test, *p* < 0.05).

For the CCA, high‐flow protocols resulted in shorter peak enhancement times regardless of contrast volume (Mann–Whitney *p* = 0.008). Within the low‐flow protocols, the low‐volume protocols showed shorter peak enhancement times than the high‐volume protocols (*p* = 0.008) (Figure 2A). For the EJV, high‐flow protocol also produced shorter peak enhancement times regardless of volume (Wilcoxon *p* = 0.043). Within low‐flow protocols, low‐volume protocol showed shorter peak enhancement time than the high‐volume protocol (*p* = 0.043) (Figure 2B). Thus, a higher injection rate significantly contributed to earlier peak enhancement times.

In the dwMRA values, the low‐flow–high‐volume protocol yielded significantly higher values compared to the low‐flow–low‐volume protocol (Mann–Whitney *p* = 0.008). The high‐flow–high‐volume protocol also showed significantly higher values than the high‐flow–low‐volume protocol (Wilcoxon *p* = 0.043). Regardless of injection rate, the high‐volume protocol yielded the widest dwMRA (Figure 2C).

There were no statistically significant differences in the maxSNR values of either the CCA or the EJV across the four protocols. Overall, CV did not differ significantly among the four protocols, but the high‐flow–high‐volume protocol showed a lower CCA CV than the high‐flow–low‐volume protocol (Wilcoxon *p* = 0.043).

### 3.2 Qualitative Assessment of Image Quality

Qualitative evaluation results are summarized in Table 4. In the qualitative assessment, the low‐flow protocol demonstrated superior temporal vessel delineation, lower venous contamination, and greater resistance to artifacts, irrespective of contrast volume.

**TABLE 4:** Qualitative assessment of parameters across four magnetic resonance angiography protocols using the time‐resolved imaging of contrast kinetics.

	Low flow–low volume (0.2 mL/s, 0.2 mL/kg)		Low flow–high volume (0.2 mL/s, 0.4 mL/kg)		High flow–low volume (2.0 mL/s, 0.2 mL/kg)		High flow–high volume (2.0 mL/s, 0.4 mL/kg)	
Parameter	Mean (rater 1, rater 2)	Median (IQR)	Mean (rater 1, rater 2)	Median (IQR)	Mean (rater 1, rater 2)	Median (IQR)	Mean (rater 1, rater 2)	Median (IQR)
Venous contamination	2 (2, 2)	2 (2–2)^a^	2.2 (2.2, 2.2)	2.5 (1.75–2.5)^a^	1.5 (1.4, 1.6)	1.5 (1.25–1.75)^a,†^	1.7 (2, 1.4)	1.5 (1–2.5)^a‡^
Temporal vessel delineation^*^	2.1 (2.4, 1.8)	2 (1.75–2.5)^a^	2.6 (2.4, 2.8)	3 (2–3)^a,†^	1.7 (2, 1.4)	2 (1.25–2)^b^	2.3 (1.8, 2.8)	2.5 (2–2.5)^a§^
Smoothness of vessel margins^*^	2.3 (2.4, 2.2)	2.5 (2–2.5)^a^	2.7 (2.4, 3)	2.5 (2.5–3)^a,†^	2.6 (2.8, 2.4)	2.5 (2.5–2.75)^a,†^	2.8 (2.8, 2.8)	3 (2.5–3 ^a§^
Ringing artifacts in the CCA	2.7 (2.6, 2.8)	3 (2.25–3)^a^	2.7 (2.8, 2.6)	2.5 (2.5–3)^a^	2.5 (2.6, 2.4)	2.5 (2–3)^a^	2.5 (3, 2)	2.5 (2.25–2.75)^a‡^
Ringing artifacts in the EJV	2.2 (2, 2.4)	2.5 (1.75–2.5)^a^	2 (1.8, 2.2)	2 (1.25–2.75)^a^	1.9 (1.8, 2)	2 (1.5–2.25)^a,†^	1.7 (1.6, 1.8)	1.5 (1.5–2)^a^
Pseudostenosis or asymmetric vascular signal loss	2.8 (2.6, 3)	3 (2.5–3)^a^	2.9 (3, 2.8)	3 (2.75–3)^a^	2.6 (2.8, 2.4)	2.5 (2.5–2.75)^a^	2.6 (2.2, 3)	2.5 (2.25–3)^a‡^

*Note*: Within each row, values with different superscript letters (a and b) indicate statistically significant differences between protocols (*p* < 0.05), as determined by the Wilcoxon signed‐rank test. Superscript symbols denote pairwise comparisons showing large effect sizes (*r* ≥ 0.5), regardless of statistical significance: ^†^ versus low‐flow–low‐volume protocol; ^‡^ versus low‐flow–high‐volume protocol; ^§^ versus high‐flow–low‐volume protocol.

In some rows, superscript letters are provided despite a non‐significant Friedman test result. These are based on post hoc Wilcoxon signed‐rank tests and should be interpreted with caution.

Abbreviations: CCA, common carotid artery; EJV, external jugular vein; IQR, interquartile range.

* Friedman test showed a significant difference among protocols (*p* < 0.05).

**FIGURE 3:**
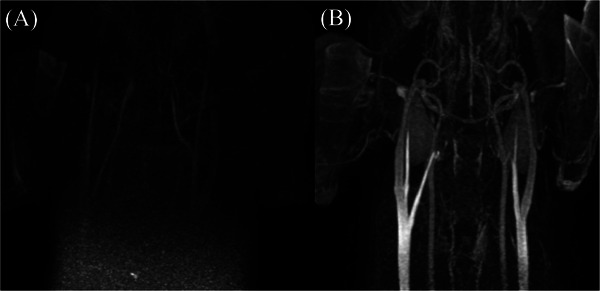
Maximum intensity projection images from the 48th phase of the time‐resolved imaging of contrast kinetics magnetic resonance angiography sequence in two dogs. (A) In a dog imaged using the low‐flow–low‐volume protocol, a notable decline in vascular contrast enhancement and signal‐to‐noise ratio was evident at the 48th phase compared to earlier phases, resulting in poor vessel conspicuity and limited differentiation from adjacent anatomical structures. (B) The other dog that underwent the low‐flow–high‐volume protocol demonstrated sustained vascular enhancement and SNR at the same phase, with clear delineation of vessels from surrounding tissues. Temporal vessel delineation scores for (A) and (B) were assigned as 1 and 3, respectively, based on qualitative assessment criteria.

In temporal vessel delineation, the low‐flow protocol showed superior delineation at low contrast volume (*p* = 0.046) and tended to provide better delineation at high volume with a larger effect size (Figure 3). Therefore, irrespective of contrast volume, the low‐flow protocol demonstrated superior delineation.

In venous contamination, although no statistically significant differences were found among the four protocols in the number of phases during which the CCA could be distinguished from the EJV, the low‐flow protocols tended to perform better than the high‐flow protocols in terms of effect size, regardless of contrast volume.

**FIGURE 4:**
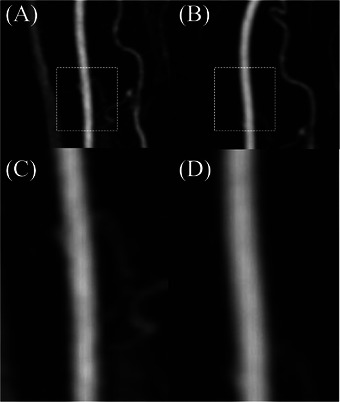
Comparison of protocol‐dependent differences in arterial wall smoothness of the common carotid artery (CCA) at peak enhancement time, obtained from time‐resolved imaging of contrast kinetics magnetic resonance angiography in a single subject. (A) CCA image at peak enhancement following the low‐flow–low‐volume protocol. (C) Enlarged view of the dotted rectangular region in (A). (B) CCA image at peak enhancement following the low‐flow–high‐volume protocol. (D) Enlarged view of the dotted rectangular region in (B). In images (A) and (C), mild irregularity or blurring of the vessel wall is observed, whereas (B) and (D) demonstrate a smooth and sharply defined vessel wall with a uniform margin. In the qualitative assessment of vessel margin smoothness, scores of 2 were assigned to (A) and (C), whereas (B) and (D) received scores of 3.

In the smoothness of vessel margins, no statistically significant pairwise differences were observed among the protocols; however, high‐volume protocols performed better regardless of injection rate, and at low contrast volume, the high‐flow protocol yielded superior results (Figure 4).

**FIGURE 5:**
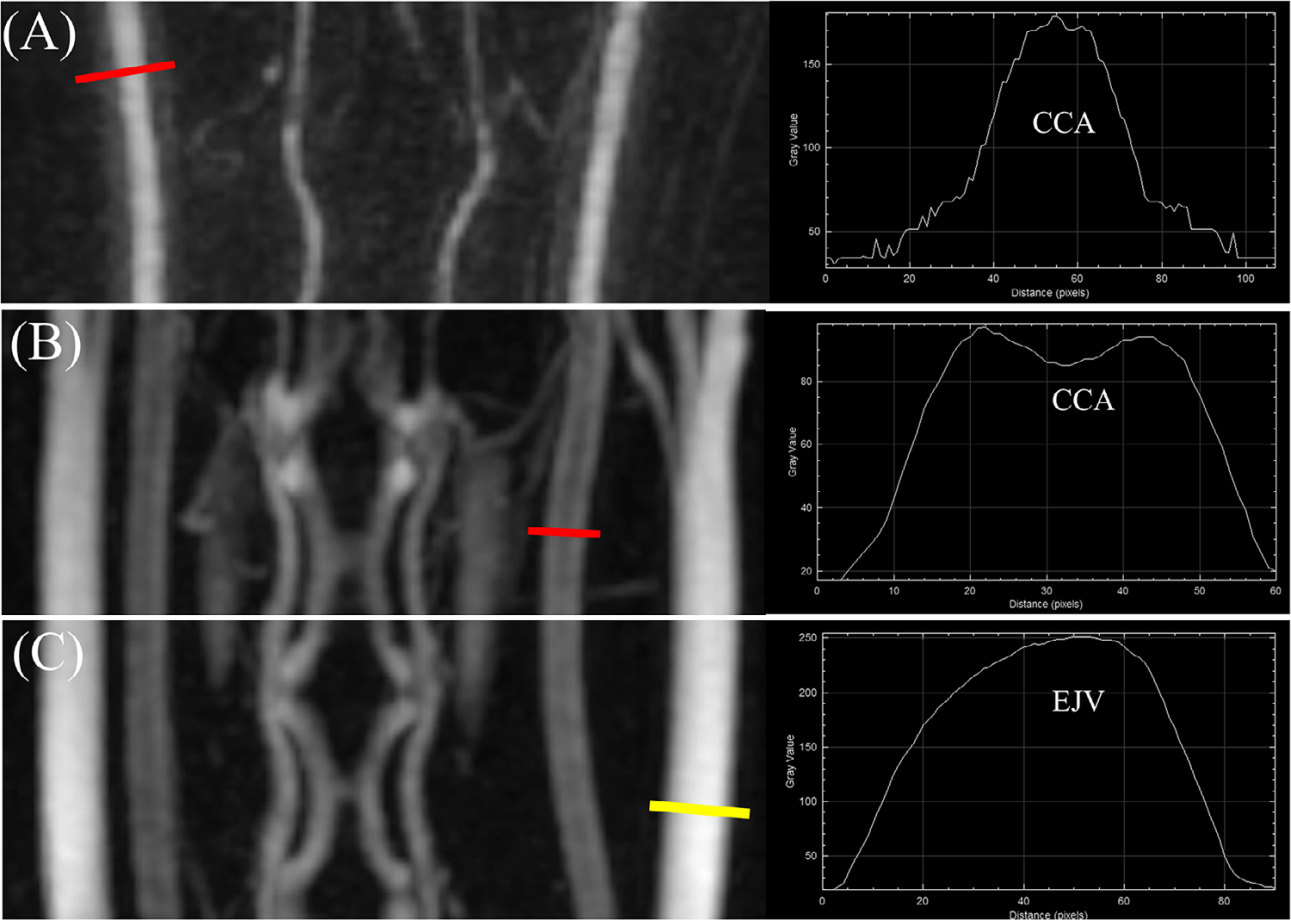
Ringing artifacts were assessed using line profile analysis on time‐resolved imaging of contrast kinetics maximum intensity projection images. In high‐flow–high‐volume protocol, a line profile drawn across the common carotid artery (CCA) demonstrated clear evidence of ringing artifact (A, red line). Similarly, in low‐flow–low‐volume protocol, the CCA also exhibited ringing artifacts along the red line profile (B), whereas the external jugular vein showed a relatively smooth signal profile without such artifacts along the yellow line (C).

**FIGURE 6:**
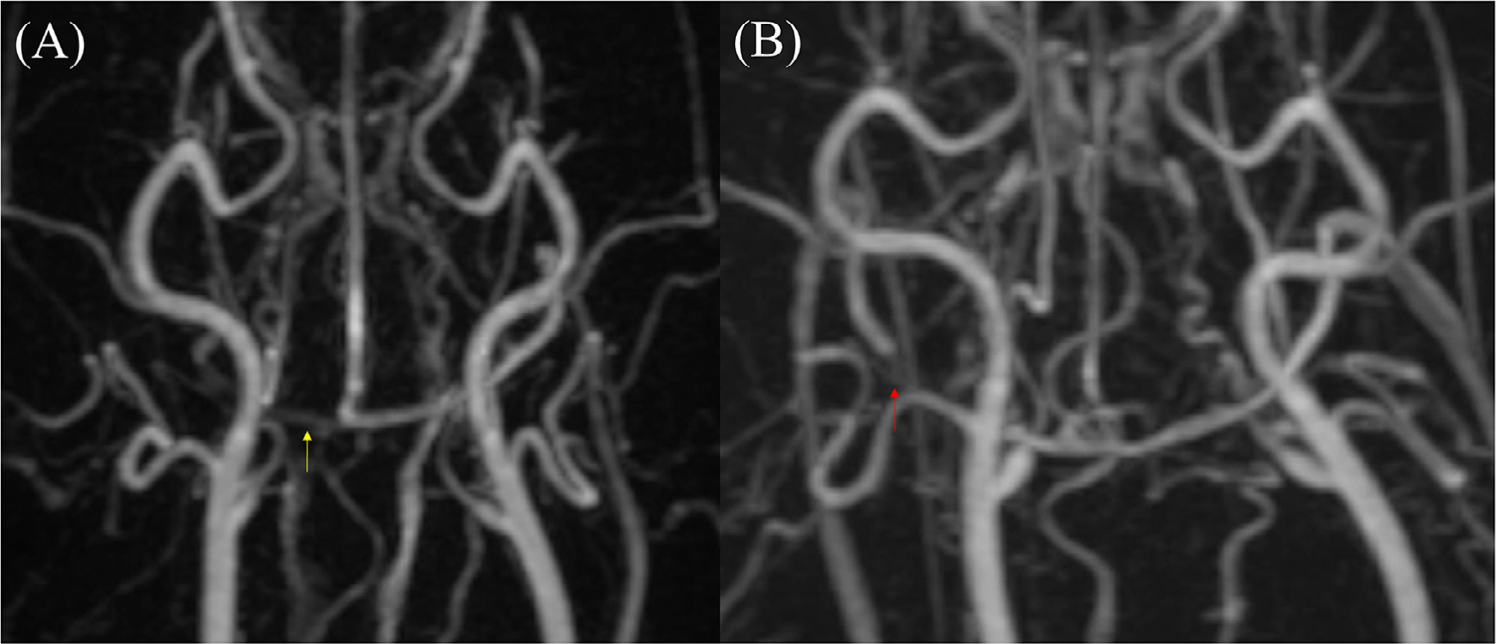
Qualitative assessment of image quality, which addresses pseudostenosis or asymmetrical vascular signal loss due to intravoxel dephasing artifacts, revealed relevant findings in two different subjects undergoing high‐flow–low‐volume protocol. In one case, the right transverse sinus exhibited marked signal reduction compared to the contralateral side (A, yellow arrow), suggestive of pseudostenosis. In another subject, partial signal loss was observed in the right temporal sinus (B, red arrow), similarly indicative of pseudostenosis. Each image was assigned a score of 2.

In image quality, which pertains to imaging artifacts, no significant differences were detected among the four protocols. Nonetheless, in terms of effect size, the low‐flow protocol tended to show better outcomes than the high‐flow protocol for both ringing artifacts (Figure 5) and intravoxel dephasing artifacts (Figure 6).

Interobserver agreement was moderate, with a weighted kappa value of 0.457 (*p* < 0.001), indicating fair to moderate reliability between the two raters for the overall qualitative scores. Agreement varied across the six qualitative assessment parameters. The weighted kappa values ranged from 0.103 to 0.681, indicating levels of agreement from slight to substantial. Substantial agreement was observed for smoothness of vessel margins (*κ* = 0.681, *p* = 0.002) and vessel delineation (*κ* = 0.540, *p* < 0.001), whereas moderate agreement was found for ringing artifacts in the EJV (*κ* = 0.394, *p* = 0.009). Venous contamination and ringing artifacts in the CCA showed fair agreement (*κ* = 0.322, *p* = 0.050 and *κ* = 0.182, *p* = 0.276, respectively), whereas pseudostenosis or asymmetric vascular signal loss demonstrated only slight agreement (*κ* = 0.103, *p* = 0.605).

### 3.3 Physiological Parameters and Scan Duration

No significant differences were observed in HR or blood pressure during anesthesia among the four protocols. The mean pre‐scan body temperature was 38.0°C ± 1.2°C (range, 36.9–38.5°C). The average mask time was 19.55 ± 2.26 s (range, 17–21 s), scan time per phase was 4.64 ± 0.5 s (range, 4.0–4.9 s), and the average total scan duration was 4 min 1 ± 27.64 s (range, 3 min 26–4 min 17 s).

## 4 Discussion

This study systematically compared four MRA protocols using the time‐resolved MRA sequence in dogs, with the aim of identifying an optimal balance between vascular visualization, temporal resolution, and artifact suppression.

In general, injection rate determines peak timing, whereas volume determines duration of enhancement [18]. With a slower but larger injection, the arterial signal is sustained longer and venous overlap is delayed, which widens the arterial window. A saline flush further helps push the bolus and reduce dispersion, making this effect more pronounced. In other words, a faster rate brings the peak earlier but shortens the window, whereas a larger volume slows the peak but extends the window. Depending on the imaging goal, adjusting rate and volume appropriately is therefore important.

Protocols with faster injection rates resulted in significantly earlier peak enhancement times for both the CCA and EJV, irrespective of volume. This result was consistent with a previous study: A canine CT study of the pancreas and liver showed significantly different peak enhancement times depending on injection duration [19]. Injection rate determines the temporal profile of contrast bolus transit: Higher rates produce a steeper arterial upslope and advance the time to peak enhancement, whereas lower rates delay peak arrival despite identical contrast volumes [10].

By contrast, the high‐volume protocol produced the widest dwMRA, indicating the most prolonged arterial‐to‐venous separation phase. This finding reflects greater temporal separation between arterial and venous enhancement and a more prolonged arterial phase with minimal venous interference, which is advantageous for vascular assessment. Accordingly, the low‐flow–high‐volume setting was identified as the most effective protocol for maintaining a prolonged arterial phase.

Unlike temporal enhancement patterns, no significant differences in maxSNR values were observed among the protocols. Thus, injection rate influenced the timing of arterial enhancement but had no clear impact on maxSNR. This finding aligns with human studies reporting that rapid injections cause earlier intravascular signal fluctuations but differs from reports that slow injections result in lower maxSNR [9]. The discrepancy may be attributable to body size differences between humans and dogs: In humans, the larger body mass provides sufficient circulation time such that even with slow injection rates—and even when the additional volume from the extension tubing prolongs the injection period—the contrast still reaches the systemic circulation gradually [10]. In dogs, however, the smaller body mass reduces this margin, and the saline contained in the extension tubing may constitute a considerable proportion of the total injection volume, thereby altering contrast dynamics. This could explain why peak enhancement time differed, whereas maxSNR remained comparable across protocols. In addition, in some veterinary studies, abdominal CT showed a comparable level of enhancement even when the contrast dose was reduced [20, 21]. This suggests that the chaser effect of saline may have contributed to the similar maxSNR observed, regardless of contrast dose. Contrast homogeneity analysis revealed that the high‐flow–high‐volume protocol achieved a lower CV for the CCA compared to the high‐flow–low‐volume protocol, indicating improved signal uniformity and potentially better vessel conspicuity when a higher contrast volume was administered at a faster rate. These results can be informative but should be interpreted with caution given the nonsignificant overall Friedman test. Nevertheless, recent human studies have demonstrated that increasing the contrast agent dose leads to stronger and more consistent vascular enhancement, with markedly reduced coefficients of variation compared to lower doses [22]. This dose–variability relationship in human studies is in line with our findings, where the high‐volume protocol produced lower CV values than the low‐volume protocol, indicating improved reproducibility of arterial enhancement.

On the basis of qualitative analysis, image quality varied depending on the MRA protocol used. The low‐flow–low‐volume protocol demonstrated significantly better persistence of arterial visibility after peak enhancement compared to the high‐flow–low‐volume protocol. Although several pairwise differences were not statistically significant, effect size analysis suggested that the low‐flow–high‐volume protocol may offer more sustained vessel visibility than the high‐flow–high‐volume protocol. In our study, rapid injection rates may have produced excessively high intravascular contrast concentrations, leading to *R*_2_ effects and intravoxel dephasing that paradoxically reduced signal. This phenomenon could partly explain why excessively rapid injections failed to maintain high *R*_1_ relaxivity and instead induced *R*_2_‐related signal loss. Therefore, when prolonged vascular conspicuity is desired following contrast administration with this sequence, a slower injection rate may be preferable.

Although venous contamination did not reach statistical significance across protocols, effect size analysis indicated relevant differences, with slow‐flow protocols demonstrating superior performance irrespective of injection volume. Several previous studies employing CT and MRI in both human and animal models have consistently shown that slower contrast injection prolongs the duration of arterial enhancement [9, 23, 24]. In contrast, the present study did not reveal statistically significant differences between protocols with respect to venous contamination but rather suggested a trend. This discrepancy may be attributable to the smaller body weight of dogs, resulting in a relatively limited initial amount of contrast medium that could not be distinctly captured by the time‐resolved MRA sequence. Furthermore, hardware constraints of the 1.5 T MRI system may have precluded the implementation of shorter scan phase times, thereby limiting temporal resolution.

Smoothness of vessel margins showed a significant overall difference across protocols. Effect size analysis revealed that higher flow rates resulted in better arterial wall delineation regardless of contrast agent volume, whereas at lower flow rates, a higher contrast volume tended to yield superior delineation. In summary, although the high‐flow–high‐volume protocol appeared to be the most favorable in terms of smoothness, this was not statistically proven.

Image quality with related artifact assessments did not reveal statistically significant differences among protocols. However, the low‐flow–high‐volume protocol tended to exhibit fewer ringing artifacts in the CCA compared to the high‐flow–high‐volume protocol, whereas the low‐flow–low‐volume protocol was less affected by venous‐phase ringing in the EJV compared to the high‐flow–low‐volume protocol. Intravoxel dephasing artifacts appeared more frequently in the high‐flow–high‐volume protocol than with the low‐flow–high‐volume protocol, suggesting that faster injection rates may increase susceptibility to phase‐related signal loss. Overall, irrespective of volume, the low‐flow protocol tended to perform better than the high‐flow protocol with respect to artifacts. Previous human studies have also shown that higher injection rates can increase ringing artifacts, as the rapid filling of the central portion of *k*‐space during steep changes in contrast concentration leads to such artifacts, which is consistent with the findings of the present study [9, 25]. However, in this study, the ringing artifacts and intravoxel dephasing appeared only in a few image phases within the affected protocols, not throughout the entire sequence. Therefore, these artifacts did not significantly reduce the overall image quality or interfere with diagnostic interpretation.

Given its comparatively favorable artifact‐related scores—including reduced ringing and dephasing—the low‐flow–high‐volume protocol appears to offer the most balanced combination of signal intensity, temporal resolution, structural clarity, and artifact suppression, and may therefore be the preferred option for time‐resolved MRA in small‐animal practice. These findings provide practical guidance for optimizing time‐resolved MRA in veterinary imaging: injection rate significantly influenced the arterial phase but not SNR; higher contrast volume enhanced vascular conspicuity; venous contamination depended more on injection rate than on volume; and slower flow tended to yield fewer artifacts—collectively supporting a protocol that combines a slow injection rate with a higher contrast volume as the most favorable among the four evaluated.

Human studies have extensively explored optimal injection parameters for CE‐MRA, and the more advanced technique, time‐resolved MRA, likewise requires intravenous contrast administration, and therefore multiple injection‐related variables such as injection rate, contrast volume, and the use of saline flushes can significantly influence image quality and diagnostic utility in the time‐resolved MRA [7, 8, 9, 26, 27]. For instance, a recent study evaluating peripheral vasculature compared injection rates of 0.5, 1.5, and 3.0 mL/s and found that a rate of 0.5 mL/s provided the best balance between reduced ringing artifacts, adequate vessel contrast, and sufficient temporal resolution [9].

In addition to protocol parameters, physiological factors, such as HR and cardiac output, can also influence contrast dynamics. Although HR was monitored before and during anesthesia, hemodynamic variability across repeated anesthetic events—performed under the same anesthetic protocol for different injection protocols—could still influence contrast distribution and signal characteristics. Given that CE‐MRA in veterinary patients is typically performed under general anesthesia, standardization or consideration of anesthetic agents and their cardiovascular effects remains important when optimizing imaging protocols.

Our study has several limitations. First, only a small number of dogs of the same breed were used. In addition, all subjects had similar body weights, which means that the results of this study may differ when applied to patients of various sizes and weights in real clinical settings. Further studies are needed to evaluate the applicability of these findings in both small dogs weighing less than 10 kg and large‐breed dogs. Second, only two injection rates, 0.2 and 2 mL/s, were assessed in this study. These limitations are due to the high cost of MRI scans, the expense of contrast agents, and a need of repeated MRI scan for comparing different MRA protocols. Third, intravascular contrast concentration can be influenced by physiological factors such as HR and cardiac output. Although HR was monitored before and during anesthesia, hemodynamic variability across repeated anesthetic events—performed under the same anesthetic protocol for different injection protocols—could still influence contrast distribution and signal characteristics. Fourth, unlike some previous studies focusing on intracranial vasculature, this study assessed contrast dynamics primarily in the CCA and EJV. The method was based on the need to optimize the time‐resolved MRA protocol for clinical application, where extracranial vessels are more accessible with larger in diameter and higher SNR. In contrast, intracranial vessels in dogs are often too small and low signal intensity for reliable quantitative analysis; thus, they were assessed only for artifact presence. In addition, compared with conventional CE‐MRA, time‐resolved MRA has inherent limitations. Its emphasis on temporal resolution comes at the expense of spatial resolution, which reduces the ability to evaluate the detailed anatomical relationships between vessels and adjacent structures. Furthermore, as a keyhole‐based technique, time‐resolved MRA relies on data‐sharing between sequential frames, which can lead to pseudo‐enhancement artifacts when bolus timing is suboptimal. These methodological limitations further highlight the importance of establishing optimized injection protocols to maximize arterial conspicuity and minimize artifacts in clinical applications. Therefore, although the present study focused on cervical vessels to optimize the protocol, further studies will be required to validate the applicability of this sequence to other anatomical regions like the pulmonary arteries, aorta, abdominal arteries, or peripheral vessels.

In conclusion, among the four time‐resolved MRA protocols evaluated, the low‐flow–high‐volume protocol (0.2 mL/s, 0.4 mL/kg) demonstrated the best balance of diagnostic window, vascular visibility, and artifact reduction. Although no significant differences were observed in maxSNR, this protocol consistently achieved superior qualitative scores and dwMRA values. The low‐flow–high‐volume protocol can be used as a practical reference for clinical and research applications of time‐resolved MRA for dogs. Further studies with larger sample sizes are warranted to validate these results.

## Author Contributions

**Category 1**

- Conception and design: Sunghwa Hong, Jihye Choi.
- Acquisition of data: Sunghwa Hong, Eunji Kim.
- Analysis and interpretation of data: Sunghwa Hong, Eunji Kim.

**Category 2**

- Drafting the article: Sunghwa Hong, Jihye Choi.
- Revising the article for intellectual content: Sunghwa Hong, Eunji Kim, Junghee Yoon, Jihye Choi.

**Category 3**

- Final approval of the completed article: Sunghwa Hong, Eunji Kim, Junghee Yoon, Jihye Choi.

**Category 4**

- Agreement to be accountable for all aspects of the work: Sunghwa Hong, Eunji Kim, Junghee Yoon, Jihye Choi.

## Conflicts of Interest

The authors declare no conflicts of interest.

## Data Availability

The data that support the findings of this study are available from the corresponding author upon reasonable request.
